# Work-Related Low Back Pain Among Physical Therapists in the Makkah Region, Saudi Arabia: A Cross-Sectional Study

**DOI:** 10.3390/healthcare13030309

**Published:** 2025-02-03

**Authors:** Hebah Ameen Takrouni, Gihan Mousa, Khalid Mohammed Yaseen, Mansour Abdullah Alshehri

**Affiliations:** 1 Department of Medical Rehabilitation Sciences, Faculty of Applied Medical Sciences, Umm Al-Qura University, Mecca 24382, Saudi Arabia; hebah.takrouni1@outlook.com (H.A.T.); gsmousa@uqu.edu.sa (G.M.); 2 Department of Physical Therapy for Cardiovascular/Respiratory Disorder and Geriatrics, Faculty of Physical Therapy, Cairo University, Giza 11432, Egypt; 3 Department of Physical Therapy, Faculty of Medical Rehabilitation Sciences, King Abdulaziz University, Jeddah 22252, Saudi Arabia; kyaseen@kau.edu.sa

**Keywords:** low back pain, musculoskeletal disorder, physical therapists, activities of daily living, work, Saudi Arabia

## Abstract

Background/Objectives: Low back pain (LBP) is a major work-related musculoskeletal disorder experienced globally, significantly limiting individuals’ daily activities and work performance. This study aimed to assess the prevalence of work-related LBP among physical therapists in the Makkah region of Saudi Arabia. Methods: A cross-sectional survey was conducted using an online self-reported questionnaire, which covered three domains: demographic information, history of LBP prior to joining the physical therapy field, and work-related LBP experienced during their current job. The questionnaire was distributed to 300 physical therapists in the Makkah region, yielding 151 responses. Data were analyzed to explore associations between LBP and various categorical and continuous factors. Results: Among the respondents, 78.1% reported experiencing LBP during their work as physical therapists, while 21.9% did not. Of those with LBP, 53.4% reported mild pain, 39.8% reported moderate pain, and smaller proportions reported severe pain (4.2%) or no pain (2.5%). Additionally, 52.5% of respondents with LBP indicated that it negatively affected their daily activities. Conclusions: Work-related LBP is highly prevalent among physical therapists in the Makkah region of Saudi Arabia, significantly impacting both patient care and the therapists’ daily functioning.

## 1. Introduction

The World Health Organization defines work-related diseases as any disease that arises as a consequence of exposure to multiple work-related risk factors [1]. Low back pain (LBP) is a debilitating health condition characterized by pain and discomfort localized below the costal margin and above the inferior gluteal folds, with or without radiating leg pain [2,3]. LBP is one of the most prevalent work-related musculoskeletal disorders globally [4], leading to significant limitations in individuals’ daily activities and occupational disabilities [5]. It can be characterized based on the duration of symptoms: acute LBP (lasting at least one day and up to six weeks), subacute LBP (pain lasting between six weeks and three months), or chronic LBP (pain persisting for more than three months) [6].

Numerous studies worldwide have reported a high prevalence of work-related musculoskeletal disorders, particularly LBP, among physical therapists due to frequent bending, lifting, stooping, turning, twisting, and prolonged sitting or standing [7,8]. Reported rates of work-related LBP among physical therapists vary across regions. These include 68.8% in Egypt [9], 46.4% in Turkey [10], and 40.1% globally [11], with specific rates of 53.4% in Africa, 50.8% in Oceania, 36.7% in Asia, 41.0% in Europe, and 33.0% in the United States. Notably, 55% of physical therapists in Kuwait [12] and 89% in Riyadh, Saudi Arabia, have reported work-related LBP [5].

LBP is a common condition with varied mechanisms. Acute LBP typically results from mechanical causes like muscle strain or disc herniation, often resolving within six weeks [13]. Chronic LBP, however, persists beyond three months and is associated with central sensitization, where the central nervous system amplifies pain signals [14]. Additionally, fear of movement can exacerbate chronic pain by promoting avoidance behaviors, contributing to deconditioning and disability [15]. For the mechanical origin of work-related LBP, key factors include prolonged sitting, poor posture, improper lifting techniques, and repetitive movements [16,17]. Additionally, sedentary work environments and inadequate ergonomic setups are prevalent causes that increase spinal strain and the risk of injury [18,19]. Understanding these factors is crucial for the effective management and treatment of LBP.

LBP is particularly common among hospital workers, especially physical therapists, and significantly impacts their professional and social lives. However, establishing work-related causes of LBP can be challenging due to the difficulty of distinguishing personal risk factors from occupational ones [20]. Given the increasing recognition of work-related LBP, further investigation is necessary to explore its prevalence and associated factors such as age, gender, physical therapy sub-specialties, and work settings. Therefore, the aim of this study was to assess the prevalence of work-related LBP among physical therapists in the Makkah region of Saudi Arabia.

## 2. Materials and Methods

### 2.1. Study Design

This cross-sectional survey study was approved by the local bioethics and medicine committee at Umm Al-Qura University (Approval No: HAPO-02-K-012-2023-01-1395). All participants provided written informed consent before participation, and de-identified data were analyzed. The participation consent form was included under the same approval number. This study adheres to the Strengthening the Reporting of Observational Studies in Epidemiology (STROBE) guidelines and includes all required reporting information.

### 2.2. Participants

The questionnaire link was distributed to 300 physical therapists and interns across Makkah, Jeddah, Taif, and neighboring cities within the Makkah region. Participants included both Saudi Physical Therapy Association (SPTA) members and non-SPTA members. A total of 151 participants responded to the survey.

Physical therapists and interns (with a minimum of 10 months of experience and at least 1 h of direct patient contact per day) were included in the study. Those working outside the Makkah region, not currently employed, or with incomplete survey responses were excluded. Other eligibility criteria included participants with LBP localized below the costal margin and above the inferior gluteal folds, with or without radiating leg pain. Both acute onset (sudden; less than 6 weeks) and chronic onset (gradual; more than three months) were considered in this study. Further information about LBP characteristics, such as pain severity and functional limitations, was collected to classify participants as having mild, moderate, or severe symptoms. Pain intensity over the past week was assessed using the Numeric Pain Rating Scale (NPRS). The classification of mild, moderate, and severe pain was based on NPRS scores, with 1–3 representing mild pain, 4–6 representing moderate pain, and 7–10 representing severe pain.

### 2.3. Questionnaire

The implemented questionnaire used for data collection in this study was developed and validated by prior studies [5,21]. This questionnaire consisted of 29 questions covering 3 main domains [5,21]: (1) personal and demographic information; (2) history of LBP prior to joining the physical therapy field; and (3) work-related LBP experienced during the participants’ current job. Additional questions addressed work setting characteristics, such as area of specialty, working hours, and other relevant factors.

### 2.4. Data Analysis

Both descriptive and inferential statistical analyses were conducted. Sociodemographic and categorical variables were summarized as frequencies and percentages, while continuous variables were presented as means and standard deviations to describe central tendency and dispersion. Results were visualized using line graphs and bar charts for improved interpretation. For inferential statistics, Fisher’s Exact Test with Monte Carlo Simulation was applied to analyze associations between categorical variables, and independent samples *t*-tests were used to assess the relationship between LBP and continuous variables. Statistical significance was set at a *p*-value < 0.05, indicating a 95% confidence interval. All analyses were performed using IBM SPSS Statistics version 27.0.1.

## 3. Results

### 3.1. Sociodemographic, Professional, and General Health Characteristics

Of the 300 physical therapists contacted, 151 participants completed the study questionnaire. The gender distribution showed that 64.9% were female and 35.1% were male. The mean age of participants was 28.7 years (SD = 6.1), with a mean height of 162.6 cm (SD = 16.1) and a mean weight of 67.5 kg (SD = 16.9). Regarding nationality, the majority were Saudi (88.7%), while 11.3% were non-Saudi. A significant portion (88.1%) reported no pre-existing medical conditions prior to developing LBP. Among those with medical conditions, 4.0% had diabetes mellitus, 2.0% had hypertension, 0.7% had cardiac diseases, and 5.3% reported other medical issues. Most participants (90.1%) had no history of medical treatment, while small percentages received hypoglycemic (3.3%), cardiac (2.0%), or antihypertensive (1.3%) medications. In terms of physical disability prior to experiencing LBP, 74.8% reported no disabilities, 23.2% had musculoskeletal issues, and 1.3% had neurological disabilities or a combination of both. The largest proportions of participants specialized in general practice (37.1%) and orthopedics (35.1%). Participants had an average of 4.9 years of experience as physical therapists (SD = 5.7). Work settings varied, with 37.7% working in general hospitals, 25.2% in private clinics, and 18.5% in private hospitals. The majority held full-time positions (82.8%), while 17.2% worked part-time, with an average of 34.9 h of direct patient contact per week. Participants were distributed across the cities of Makkah (39.7%), Taif (29.1%), and Jeddah (29.1%). Additionally, 35.8% were members of SPTA, while 64.2% were not (Table 1).

### 3.2. LBP Before Working as a Physical Therapist

Regarding LBP experienced before entering the physical therapy profession, 61.6% of participants reported no prior LBP, while 31.1% experienced mild LBP, 6.6% reported moderate LBP, and only 0.7% reported severe LBP. In terms of functional limitations due to LBP before joining the profession, 76.8% reported no limitations, 15.9% experienced mild limitations, 6.6% reported moderate limitations, and 0.7% had severe limitations (Table 2).

### 3.3. LBP During Work as a Physical Therapist

**Figure 1:**
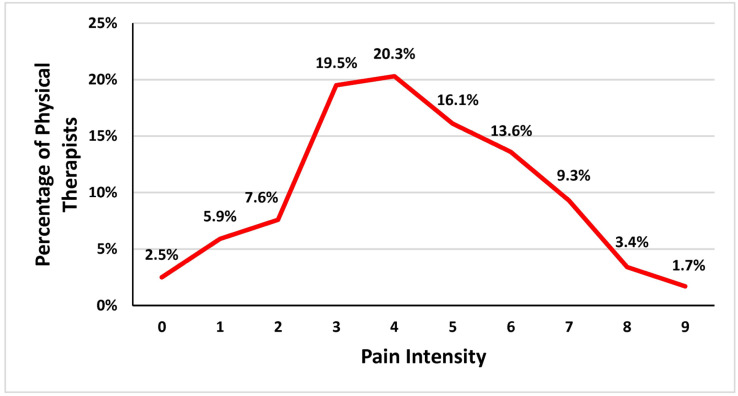
Rating score of pain experienced during work as a physical therapist.

**Figure 2:**
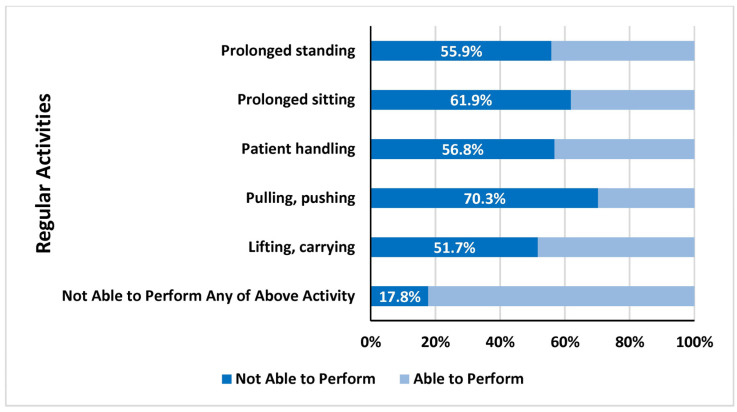
Performance of regular activities due to LBP during work as a physical therapist.

**Figure 3:**
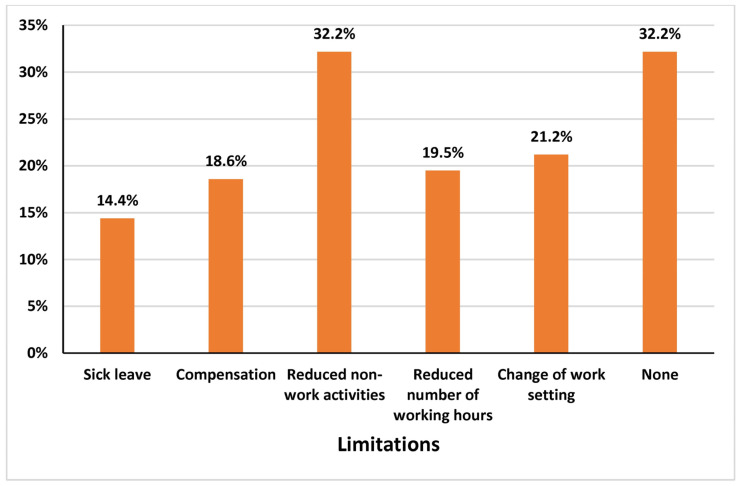
Limitations due to LBP related to work as a physical therapist.

**Figure 4:**
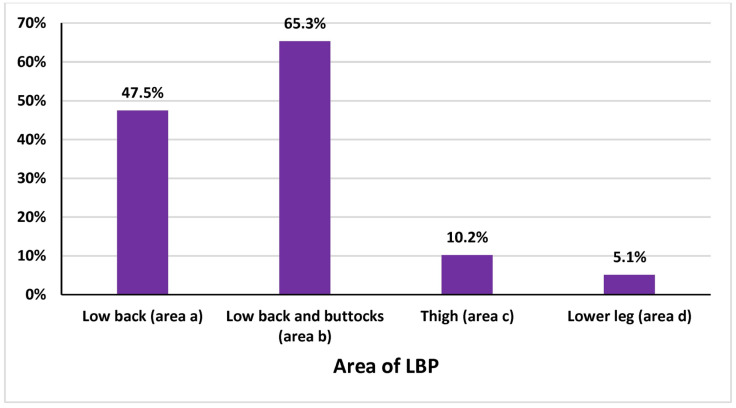
Area of LBP related to work as a physical therapist.

Among the participants, 78.1% reported experiencing LBP during their work as physical therapists, while 21.9% did not. Among those who experienced LBP, 53.4% reported mild pain, 39.8% reported moderate pain, and smaller proportions reported severe pain (4.2%) or no pain (2.5%) (Table 3). The onset of LBP was reported as acute (sudden) by 43.2% of participants and chronic (gradual) by 56.8%. The duration of pain episodes varied: 63.6% experienced pain lasting 1 week, 21.2% reported pain lasting 2 to 4 weeks, and 15.3% experienced pain for more than 4 weeks. Work settings where injuries occurred included hospitals (60.2%), private clinics (27.1%), community care settings (5.1%), universities (5.1%), and special schools (2.5%) (Table 3). Among those who experienced LBP, 52.5% reported that LBP affected their regular activities. Furthermore, 61.9% of participants reported that they were still experiencing LBP at the time of the study. Pain intensity scores were varied and are visualized in Figure 1. Figure 2, Figure 3 and Figure 4 further illustrate participants’ performance of regular activities, limitations due to LBP, and the specific areas of LBP, respectively. These data might provide a further understanding of the prevalence, characteristics, and impact of work-related LBP among physical therapists in Saudi Arabia, which can help in planning suitable and effective preventive measures and interventions.

### 3.4. Factors Associated with LBP During Work as a Physical Therapist

The factors associated with LBP during work were analyzed in relation to sociodemographic, professional, and general health characteristics. Using Fisher’s Exact Test with Monte Carlo Simulation and independent samples *t*-tests, the results indicated no statistically significant associations between LBP and variables such as gender (*p* = 0.409), age (*p* = 0.976), height (*p* = 0.524), weight (*p* = 0.733), nationality (*p* = 1.000), medical conditions prior to LBP (*p* = 0.562), past medical treatment (*p* = 0.912), and physical disabilities before LBP (*p* = 0.098). Work-related factors, including area of specialty, years of experience, work setting, nature of employment, number of hours of direct patient contact per week, SPTA membership, and city of practice, also showed no significant associations with LBP (all *p*-values > 0.05) (Table 4).

## 4. Discussion

This study hypothesized a significant prevalence of LBP among physical therapists working in the Makkah region of Saudi Arabia. The findings revealed a 78.1% prevalence of LBP among participants, with 61.9% reporting current (present) LBP, regardless of gender or pain intensity.

These results align with previous studies. For instance, Abolfotouh et al. [2] reported a 73% prevalence of LBP in the past 12 months among participants, and Shehab et al. [22] found a 70% prevalence of LBP among physical therapists in Kuwait, with 57% experiencing current LBP. Although the current study did not find a statistically significant association between work settings and LBP, general hospitals exhibited the highest prevalence (77.2%) in numbers, followed by private clinics (73.7%). The higher prevalence of LBP among physiotherapists working in general hospitals may be attributed to the demanding nature of their work, which often involves handling in-patients with complex conditions and managing higher patient loads, including both in-patient and out-patient care. These factors, combined with physically taxing tasks such as lifting, repositioning patients, and prolonged standing, may contribute to an increased risk of LBP. In contrast, physiotherapists working in private clinics generally experience a lower workload and fewer physical demands. Similar findings have been reported among healthcare providers, such as nurses at King Abdulaziz University Hospital (65.7%) and medical and surgical residents at King Abdulaziz Medical City in Riyadh (53%) [23,24].

LBP is often assumed to be associated with physically demanding tasks, such as patient handling, lifting, carrying, pulling, pushing, prolonged standing, and sitting. However, these activities were not significant pain triggers for participants in this study, which corroborates the results of Alghadir et al. [5]. Newly graduated or less experienced physical therapists may be at greater risk due to inadequate knowledge of protective techniques and ergonomics [25,26]. Jensen et al. [27] similarly noted that female healthcare providers with no prior history of LBP but with high physical work demands were at increased risk for LBP.

To manage the impact of LBP, some physical therapists in this study adapted by reducing non-work activities, modifying their work settings, or altering their working strategies. However, 32.2% reported no limitations or changes in response to their condition [28]. Other studies have shown that LBP affects social activities and professional responsibilities, such as meetings, resulting in reduced productivity and increased absenteeism [29]. LBP imposes a significant economic burden on individuals and society, driven by treatment costs, lost work hours, and decreased productivity [30,31]. Therefore, physiotherapists should be encouraged to report workplace injuries, and barriers to official reporting must be addressed.

The study was limited to one region of Saudi Arabia. To achieve more comprehensive results, future studies should include larger sample sizes and expand to other regions, such as Al Madinah, or include additional cities within the Makkah region. Additionally, the study did not collect data on specific musculoskeletal conditions that participants may have had, which may limit the ability to fully assess their role as confounding factors in the relationship between work-related activities and LBP. Future research should investigate the prevalence of work-related musculoskeletal disorders in other regions of the body and focus on identifying and implementing strategies to alleviate LBP, especially in high-risk professions like physical therapy. Education and training programs should emphasize both theoretical and practical knowledge of ergonomics and safe patient-handling techniques, which can be facilitated through workplace initiatives and educational committees. Employers are encouraged to introduce workplace programs that incorporate non-work activities such as Tai Chi, swimming, and aerobic exercises to enhance job satisfaction, reduce stress, and improve overall physical well-being.

## 5. Conclusions

Physical therapists in the Makkah region of Saudi Arabia demonstrated a significant prevalence of work-related LBP. This might be considered one of the factors that could negatively impact the care delivered to patients, as well as the therapists’ performance. However, this requires further investigation.

## Figures and Tables

**Table 1:** Demographic, professional characteristics, and general health history of participants.

Items		N	%	Mean	SD
Gender	Female	98	64.9		
	Male	53	35.1		
Age				28.7	6.1
Height (cm)				162.6	16.1
weight (Kg)				67.5	16.9
Nationality	Saudi	134	88.7		
	Non-Saudi	17	11.3		
Medical disease before developing LBP	None	133	88.1		
	Others	8	5.3		
	Diabetes mellitus	6	4.0		
	Hypertension	3	2.0		
	Cardiac diseases	1	0.7		
Past medical treatment conditions	None	136	90.1		
	Others	5	3.3		
	Hypoglycemic (oral, insulin)	5	3.3		
	Cardiac medications	3	2.0		
	Antihypertensive	2	1.3		
Physical disability prior to developing LBP	None	113	74.8		
	Musculoskeletal	35	23.2		
	Neurological	2	1.3		
	Both	1	0.7		
Area of specialty	General practice	56	37.1		
	Orthopedics	56	37.1		
	Pediatrics	11	7.3		
	Neurology	9	6.0		
	Home care	6	4.0		
	Geriatrics	5	3.3		
	Cardiology	5	3.3		
	Others (e.g., sports, acute care)	3	2.0		
Years of experience as a physical therapist				4.9	5.7
Work setting	General hospital	57	37.7		
	Private clinic	38	25.2		
	Private hospital	28	18.5		
	Rehabilitation hospital	12	7.9		
	Community care	9	6.0		
	Special schools	4	2.6		
	University	3	2.0		
Nature of job	Full-time physical therapist	125	82.8		
	Part-time	26	17.2		
Number of hours of direct patient contact per week				34.9	11.63
City	Makkah	60	39.7		
	Taif	44	29.1		
	Jeddah	44	29.1		
	Rabigh	2	1.3		
	Al-Qunfudhah	1	0.7		
Member of Saudi Physical Therapy Association	No	97	64.2		
	Yes	54	35.8		

N: frequency; %: percentage; SD: standard deviation; LBP: low back pain.

**Table 2:** History of LBP before working as a physical therapist.

Items		N	%
Before working as a physical therapist, what type of LBP did you suffer?	None	93	61.6
	Mild	47	31.1
	Moderate	10	6.6
	Severe	1	0.7
Before working as a physical therapist what type of functional limitations did you experience as a result of LBP?	None	116	76.8
	Mild	24	15.9
	Moderate	10	6.6
	Severe	1	0.7

LBP: low back pain; N: frequency; %: percentage.

**Table 3:** History of LBP during work as a physical therapist.

Items		N	%
Did you have LBP during your work as a physical therapist?	No	33	21.9
	Yes	118	78.1
If yes (N = 118), during work as physical therapist what type of LBP have you experienced?	Mild	63	53.4
	Moderate	47	39.8
	None	3	2.5
	Severe	5	4.2
Onset of pain/injury	Acute (sudden)	51	43.2
	Chronic (gradual)	67	56.8
Average duration of pain in days during LBP episode	>4 weeks	18	15.3
	1 week	75	63.6
	2–4 weeks	25	21.2
Work setting in which injury occurred	Hospital	71	60.2
	Private	32	27.1
	Community care	6	5.1
	University	6	5.1
	Special school	3	2.5
Have you been affected by LBP from doing your regular activities?	No	56	47.5
	Yes	62	52.5
Are you currently (presently) experiencing LBP?	No	45	38.1
	Yes	73	61.9

LBP: low back pain; N: frequency; %: percentage.

**Table 4:** Association of LBP during work as a physical therapist with sociodemographic, professional characteristics, and general health.

Items		Experienced LBP During Work as a Physical Therapist				
		No		Yes		*p*-Value
		N	%	N	%	
Gender	Female	19	19.4	79	80.6	0.409
	Male	14	26.4	39	73.6	
Age		28.7	6.1	28.7	6.2	0.976
Height (cm)		160.0	29.8	163.3	9.4	0.524
weight (Kg)		66.6	16.9	67.7	17.0	0.733
Nationality	Saudi	30	22.4	104	77.6	1.000
	Non-Saudi	3	17.6	14	82.4	
Medical disease before developing LBP	None	32	24.1	101	75.9	0.562
	Others	0	0.0	8	100.0	
	Diabetes mellitus	1	16.7	5	83.3	
	Hypertension	0	0.0	3	100.0	
	Cardiac diseases	0	0.0	1	100.0	
Past medical treatment conditions	None	32	23.5	104	76.5	0.912
	Others	0	0.0	5	100.0	
	Hypoglycemic (oral, insulin)	1	20.0	4	80.0	
	Cardiac medications	0	0.0	3	100.0	
	Antihypertensive	0	0.0	2	100.0	
Physical disability prior to developing LBP	None	30	26.5	83	73.5	0.098
	Musculoskeletal	3	8.6	32	91.4	
	Neurological	0	0.0	2	100.0	
	Both	0	0.0	1	100.0	
Area of specialty	General practice	10	17.9	46	82.1	0.466
	Orthopedics	14	25.0	42	75.0	
	Pediatrics	4	36.4	7	63.6	
	Neurology	0	0.0	9	100.0	
	Home care	2	33.3	4	66.7	
	Geriatrics	1	20.0	4	80.0	
	Cardiology	1	20.0	4	80.0	
	Others (e.g., sports, acute care)	1	33.3	2	66.7	
Years of experience as a physical therapist		5.0	6.0	4.9	5.6	0.967
Work setting	General hospital	13	22.8	44	77.2	0.828
	Private clinic	10	26.3	28	73.7	
	Private hospital	6	21.4	22	78.6	
	Rehabilitation hospital	1	8.3	11	91.7	
	Community care	1	11.1	8	88.9	
	Special schools	1	25.0	3	75.0	
	University	1	33.3	2	66.7	
Nature of job	Full-time physical therapist	27	21.6	98	78.4	1.000
	Part-time	6	23.1	20	76.9	
Number of hours of direct patient contact per week		32.6	11.1	35.5	11.8	0.193
City	Makkah	13	21.7	47	78.3	0.816
	Taif	12	27.3	32	72.7	
	Jeddah	8	18.2	36	81.8	
	Rabigh	0	0.0	2	100.0	
	Al-Qunfudhah	0	0.0	1	100.0	
Member of Saudi Physical Therapy Association	No	23	23.7	74	76.3	0.541
	Yes					
		10	18.5	44	81.5	

LBP: low back pain; N: frequency; %: percentage.

## Data Availability

The original contributions presented in the study are included in the article; further inquiries can be directed at the corresponding author.
